# Arsenic in drinking water and urinary tract cancers: a systematic review of 30 years of epidemiological evidence

**DOI:** 10.1186/1476-069X-13-44

**Published:** 2014-06-02

**Authors:** Nathalie Saint-Jacques, Louise Parker, Patrick Brown, Trevor JB Dummer

**Affiliations:** 1 Cancer Care Nova Scotia, Surveillance and Epidemiology Unit, Room 560 Bethune Building, 1276 South Street, Halifax B3H 2Y9, Nova Scotia, Canada; 2 Interdisciplinary PhD program, Dalhousie University, 6299 South Street, Room 314, PO Box 15000, Halifax B3H 4R2, Nova Scotia, Canada; 3 Department of Pediatrics and Population Cancer Research Program, Dalhousie University, 1494 Carlton Street, PO Box 15000, Halifax B3H 4R2, Nova Scotia, Canada; 4 Population Studies and Surveillance, Cancer Care Ontario, 620 University Ave, Toronto M5G 2 L7 Ontario, Canada

**Keywords:** Arsenic, Drinking water, Bladder, Kidney, Urinary tract, Cancer risk, Systematic review, Meta-analysis

## Abstract

**Background:**

Arsenic in drinking water is a public health issue affecting hundreds of millions of people worldwide. This review summarizes 30 years of epidemiological studies on arsenic exposure in drinking water and the risk of bladder or kidney cancer, quantifying these risks using a meta-analytical framework.

**Methods:**

Forty studies met the selection criteria. Seventeen provided point estimates of arsenic concentrations in drinking water and were used in a meta-analysis of bladder cancer incidence (7 studies) and mortality (10 studies) and kidney cancer mortality (2 studies). Risk estimates for incidence and mortality were analyzed separately using Generalized Linear Models. Predicted risks for bladder cancer incidence were estimated at 10, 50 and 150 μg/L arsenic in drinking water. Bootstrap randomizations were used to assess robustness of effect size.

**Results:**

Twenty-eight studies observed an association between arsenic in drinking water and bladder cancer. Ten studies showed an association with kidney cancer, although of lower magnitude than that for bladder cancer. The meta-analyses showed the predicted risks for bladder cancer incidence were 2.7 [1.2–4.1]; 4.2 [2.1–6.3] and; 5.8 [2.9–8.7] for drinking water arsenic levels of 10, 50, and 150 μg/L, respectively. Bootstrapped randomizations confirmed this increased risk, but, lowering the effect size to 1.4 [0.35–4.0], 2.3 [0.59–6.4], and 3.1 [0.80–8.9]. The latter suggests that with exposures to 50 μg/L, there was an 83% probability for elevated incidence of bladder cancer; and a 74% probability for elevated mortality. For both bladder and kidney cancers, mortality rates at 150 ug/L were about 30% greater than those at 10 μg/L.

**Conclusion:**

Arsenic in drinking water is associated with an increased risk of bladder and kidney cancers, although at lower levels (<150 μg/L), there is uncertainty due to the increased likelihood of exposure misclassification at the lower end of the exposure curve. Meta-analyses suggest exposure to 10 μg/L of arsenic in drinking water may double the risk of bladder cancer, or at the very least, increase it by about 40%. With the large number of people exposed to these arsenic concentrations worldwide the public health consequences of arsenic in drinking water are substantial.

## Background

Arsenic (As) is a naturally occurring toxic metalloid prevalent in the earth’s crust [1]. It enters drinking-water sources in a dissolved state primarily resulting from the weathering of rocks [2]. Human exposure to As involve multiple pathways [3-9], with drinking water being the primary route of exposure for the majority of highly exposed populations [4,9,10]. West Bengal, Bangladesh and Taiwan are the most affected regions worldwide [4,11-14]. In these areas, As concentration as high as 4,700 μg/L have been reported in drinking water, and levels in excess of 300 μg/L are common. High levels of As in drinking water have also been reported elsewhere, such as North and South America, Central and Eastern Europe as well as Australia [4,11,15-22].

The contamination of drinking water by As has become an ongoing public health issue affecting hundreds of millions of people worldwide. A growing body of evidence supporting a wide range of acute and chronic effects on health, including cancer [5,20-72], has led the World Health Organization (WHO) to lower the advisory limit for concentration of As in drinking water from 25 μg/L to a provisional guideline limit of 10 μg/L [10]. However, many developing countries continue to endorse an effective upper limit of 50 μg/L [4].

The International Agency for Research on Cancer (IARC) has classified inorganic As in drinking water as a Group 1 carcinogen [73]. Suggested mechanisms of action for As carcinogenesis include oxidative damage, epigenetic effects and interference with DNA repair, mechanisms which have been specifically implicated in the development of As-related urinary tract cancers which are the focus of this review [74-81]. Urinary tract cancers comprise primarily cancers of the urinary bladder and kidney, the former being the ninth most common cause of cancer worldwide [82]. Most studies generally report on bladder or kidney cancer, although some of the studies included in this review and meta-analysis reported histologies, mostly urothelial/transitional cell and renal cell carcinomas. Tobacco smoking and most notably, the ingestion of high levels of inorganic As are two important risk factors for bladder and kidney cancers [83-86].

To date, epidemiological studies of populations exposed to high levels of inorganic As have shown strong associations and dose–response relationships between As in drinking water and bladder cancer and; potential associations with kidney cancer [23]. Typically, these studies report on areas of extreme exposure where levels of As in drinking water range from 150 to over 1000 ug/L. The extent to which health effects may develop remain uncertain at lower levels of exposure (< 150 μg/L), with many studies failing to demonstrate the risk that might be expected by extrapolation from findings related to high levels of exposure [5].

This paper reviews findings from epidemiological studies published over the past 30 years, including a number of recent publications focusing on low-levels exposure and bladder and kidney cancer outcomes [60,63,67,87]. It also quantifies the risk of urinary tract cancers due to exposure to As in drinking water, combining risk estimates from published epidemiological data. As such, this work complements the recent systematic review of IARC which reports on carcinogenicity following exposure to As [23].

Most studies reporting on urinary cancers risk and As exposure tend to focus on specific levels of exposure. By combining exposure levels from multiple studies, the review profiles a more complete and continuous range of As exposure from which to better assess and predict cancer risks associated with varying levels of exposure. This meta-analytical approach is especially relevant to shed light on dose–response relationship, especially at the lower end of the curve where there has been the most uncertainty and where a large number of people may be at risk.

## Methodology

### Review process

Searches of the Medline (PubMed) and Embase databases were conducted to identify studies reporting on exposure to As in drinking water and urinary tract cancer outcomes and published prior to January 2013. The search conditions are presented in Table 1. Searches were also undertaken using Google Scholar and the WHO and the IARC publications [3,23]. Studies were selected based on the selection criteria listed in Table 1. Information abstracted from reviewed articles is shown in Tables 2, 3, 4, 5, 6. When the distribution of As in drinking water was detailed in another publication, that information was also retrieved. Where available, the adjusted relative risks estimates and associated 95% confidence intervals were selected.

**Table 1:** Search conditions and criteria for study selection

**Search conditions**	**Study selection**
((arsenic) AND ("bladder cancer*" OR "kidney cancer*" OR "urinary tract cancer*" OR "upper urinary tract cancer*" OR "urinary tract cancer*" OR "urologic neoplasm*" OR "cancer*, urinary tract" OR "kidney neoplasm*" OR "carcinoma, renal cell*" OR "urinary bladder neoplasm*" OR "urinary tract disease*" OR "kidney tumour*" OR "bladder tumour*" OR "bladder tumor*"OR "kidney tumor*" OR renal cell* carcinoma” OR "bladder neoplasms") AND ("water" OR "drinking water" OR "water supply" OR "toenail" OR "urine" OR "well water") ^†^	1. Arsenic in drinking water, toenail or urine, as exposure of primary interest.
	2. Urinary tract cancers incidence and mortality as primary outcome.
	3. Original study that published the data.
	4. Relative risk estimates, measures of variability (i.e., confidence intervals) documented.
	5. Epidemiological study designs, including ecological, case-control or cohort study.
	6. English language publications.

^†^The wildcard (*) was used to identify any other characters.

**Table 2:** Summary results from ecological studies reporting on arsenic exposure and the risk of bladder cancer

**Study [reference] (Table from original publication)**	**Study locale**	**Outcome**	**Exposure**^**1**^**[comments]**	**ICD**^**2**^	**Outcome measure**	**Cases**	**Risk estimate (95% CI)**
Chen et al. 1985^3^[24]	84 villages from 4 neighbouring townships on SW coast, Taiwan	Mortality 1968-82	Median arsenic content of artesian well and (range): 780 μg˙•L^-1^ (350–1,140); in shallow well: 40 (0.0–300). Period of samples collection not reported.	ICD 188	SMR_male_	167	11.0 (9.33–12.7)
					SMR_female_	165	20.1 (17.0–23.2)
			[Comparison of mortality rate in Blackfoot disease-endemic areas (BFD) with those of the general population.]				
*Chen et al. 1988^4^[26] (Table One)	BFD endemic area, Taiwan	Mortality 1973-86	Arsenic well water concentration (μg˙•L^-1^). Period of samples collection not reported.	ICD9 188			
			General population		ASMR_male_		
			< 300			–	3.1
			300-590			–	15.7
			≥ 600			–	37.8
						–	89.1
			General population		ASMR_female_		
			< 300			–	1.4
			300-590			–	16.7
			≥ 600			–	35.1
			[Comparison of mortality rate in BFD with those of the general population.]			–	91.5
*Wu et al. 1989^5^[27] (Table Three)	BFD endemic area, Taiwan (42 villages)	Mortality 1973-86	Arsenic well water concentration (μg˙•L^-1^) based on well water samples collected between 1964–66.	ICD8 188			
			< 300		ASMR_male_	23	22.6
			300–590			36	61.0
			≥ 600			26	92.7
			< 300		ASMR_female_	30	25.6
			300–590			36	57.0
			≥ 600			30	111.3
Chen and Wang 1990^6^[28] (Table Four)	314 precincts & townships in Taiwan, including 4 from BFD endemic area	Mortality 1972-83	Average arsenic levels in water samples of all 314 geographical units. 73.9% had < 5% of wells with > 50 μg˙•L^-1^ ; 14.7% had 5-14%; 11.5% had ≥ 15%. Well water samples collected between 1974–76.	ICD 188			
			All precincts & townships		ASMR_male_	–	3.9 (0.5)
					ASMR_female_	–	4.2 (0.5)
			Southwestern townships		ASMR_male_	–	3.7 (0.7)
					ASMR_female_	–	4.5 (0.7)
Chiang et al. 1993^7^[29] (Table Two)	BFD endemic area in Taiwan and 2 neighbouring areas	Incidence 1981-85	Exposure not evaluated, but based on Chen et al. 1985, the median arsenic content of artesian well in this area was 780 μg˙•L^-1^ (350 – 1,140); that of shallow well was 40 μg˙•L^-1^ (0.0 – 300). Period of samples collection not reported.	N/A	Endemic area		
					IR__both_sex_	140	23.5
					IR_male_	81	26.1
					IR_female_	59	21.1
			[Comparison of incidence rate in BFD with those of neighbouring areas and Taiwan as a whole.]		Neighbouring Endemic area		
					IR__both_sex_	13	4.45
					IR_male_	7	4.65
					IR_female_	6	4.28
					All Taiwan		
					IR__both_sex_	2,135	2.29
					IR_male_	1,608	3.31
					IR_female_	527	1.17
Hopenhayn-Rich et al. 1996^8^[35] (Table Three)	26 counties in Cordoba, Argentina	Mortality 1986-91	Arsenic drinking water concentration ranging from 100 to 2,000 μg˙•L^-1^.	ICD9 188			
*Hopenhayn-Rich et al. 1998 [36] (Tables Three, Four)			Low			113	0.80 (0.66–0.96)
			Medium		SMR_male_	116	1.28 (1.05–1.53)
			High (178 μg˙•L^-1^ on average)			131	2.14 (1.78–2.53)
			Low			39	1.21 (0.85–1.64)
			Medium		SMR_female_	29	1.39 (0.93–1.99)
			High (178 μg˙•L^-1^ on average)			27	1.82 (1.19–2.64)
			[Arsenic measurements from a variety of sources, including official reports of water analyses from the 1930, 2 scientific sampling studies and a water survey.]				
Guo et al. 1997^9^[37] (Table Two)	243 townships in Taiwan	Incidence 1980-87	Arsenic well water concentration ranging from < 50 to > 640 μg˙•L-^1^.	ICD 188	RD_male_	–	0.57 (0.07)
			Estimate presented measured at > 640 μg˙•L^-1^.		RD_female_	–	0.33 (0.04)
			[Arsenic measurements from a National survey of 83,656 wells in 243 townships, collected mostly between 1974–76.]				
Rivara et al.1997 [38] (Table Four)	Chile	Mortality 1950-92	Annual average arsenic concentration in drinking water for Antofagasta (Region II of Chile) ranging between 40 to 860 μg˙•L^-1^. Data from historical records from 1950–1992.	ICD 188	RR	–	10.2 (8.6–12.2)
			[Comparison of mortality rate in Region II (exposed populations) vs Region VIII (control populations.]				
Smith et al. 1998 [39]	Chile	Mortality 1989-93	Region II of Northern Chile with population weighted average arsenic concentration in drinking water up to 569 μg˙•L^-1^ compared with the rest of Chile; exposure generally < 10 μg˙•L^-1^.	N/A	SMR_male_	93	6.0 (4.8–7.4)
					SMR_female_	64	8.2 (6.3–10.5)
			[Arsenic measurements from 1950–94.]				
Hinwood et al. 1999 [88] (Table Two)	22 areas in Victoria, Australia	Incidence 1982-91	Median water arsenic concentration ranging 13 μg˙•L^-1^ to 1,077 μg˙•L^-1^.	ICD 188, 189.1-189.3	SIR	303	0.94 (0.84–1.06)
			[Selected areas were those where samples with soil and/or water arsenic concentration were generally in excess of 10 μg˙•L^-1^. Period for samples collection is not available.]				
*Tsai et al. 1999 [41] (Tables Two, Three)	4 townships from BFD endemic area in SW coast, Taiwan	Mortality 1971-94	Median arsenic content of artesian well: 780 μg˙•L^-1^ (range: 350–1,140). Period of samples collection not reported. Authors state that artesian wells were no longer used by the mid-1970s.	ICD9 188	SMR_local-male_	312	8.92 (7.96–9.96)
					SMR_national-male_	312	10.5 (9.37–11.7)
			[Comparison of mortality in BFD endemic area with that of a local reference population (Chiayi-Tainan county) and that of Taiwan as a whole.]		SMR_local-female_	295	14.1 (12.51–15.8)
					SMR_national-female_	295	17.8 (5.70–19.8)
*Lamm et al. 2004^10^[89] (Table One)	133 counties in 26 states, USA	Mortality 1950-79	Arsenic groundwater water concentration (μg˙•L^-1^). Period of samples collection not reported.	N/A		Counties	
			3.0–3.9		SMR_white_male_	53	0.95 (0.89–1.01)
			4.0–4.9		SMR_white_male_	22	0.95 (0.88–1.02)
			5.0–7.4		SMR_white_male_	28	0.97 (0.85–1.12)
			7.5–9.9		SMR_white_male_	14	0.89 (0.75–1.06)
			10.0–19.9		SMR_white_male_	11	0.90 (0.78–1.04)
			20.0–49.9		SMR_white_male_	3	0.80 (0.54–1.17)
			50.0–59.9		SMR_white_male_	2	0.73 (0.41–1.27)
			[Median arsenic concentration ranged between 3–60 (μg˙•L^-1^), with 65% of the counties and 82% of the population in the range of 3–5 (μg˙•L^-1^).]				
Marshall et al. 2007 [50] (Table Three)	Chile	Mortality 1950-2000	Northern Chile (Region II) with population weighted average arsenic concentration in drinking water up to 569 μg˙•L^-1^ vs Region V which is otherwise similar to Region II but not exposed to arsenic. Between 1958–1970, arsenic concentration in water supply of Antofagasta and nearby Mejillones (Region II) averaged 870 μg˙•L^-1^ and declined in the 1970s when water treatment plants were installed.	ICD 188			
					RR_male-1971–73_	9	1.71 (0.80–3.69)
					RR_male-1974–75_	9	5.95 (2.22–16.0)
					RR_male-1977–79_	17	2.10 (1.19–3.72)
					RR_male-1980–82_	35	5.04 (3.13–8.10)
					RR_male-1983–85_	41	5.77 (3.66–9.09)
					RR_male-1986–88_	47	6.10 (3.97–9.39)
					RR_male-1989–91_	52	4.73 (3.23–6.94)
					RR_male-1992–94_	62	4.95 (3.47–7.06)
					RR_male-1995–97_	56	4.43 (3.07–6.38)
					RR_male-1998–2000_	58	4.27 (2.98–6.11)
					RR_female-1971–73_	7	3.45 (1.34–8.91)
					RR_female-1974–75_	4	3.09 (0.90–10.6)
					RR_female-1977–79_	10	5.39 (2.24–13.0)
					RR_female-1980–82_	22	9.10 (4.59–18.1)
					RR_female-1983–85_	22	8.41 (4.30–16.4)
					RR_female-1986–88_	37	7.28 (4.44–12.0)
					RR_female-1989–91_	35	6.61 (4.02–10.9)
					RR_female-1992–94_	42	13.8 (7.74–24.5)
					RR_female-1995–97_	44	7.60 (4.78–12.1)
					RR_female-1998–2000_	50	9.16 (5.76–14.5)
*†Meliker et al. 2007 [90] (Table Two)	6 counties, Southeastern Michigan, USA	Mortality 1979-97	Population weighted median arsenic concentration in water of 7.58 μg˙•L^-1^. Data from 9,251 well water samples collected between 1983–2002.	ICD9 188	SMR_male_	348	0.94 (0.82–1.08)
					SMR_female_	171	0.98 (0.80–1.19)
*†Pou et al. 2011^12^[63] (Table Two)	26 counties in province of Cordoba, Argentina	Mortality 1986-2006	Arsenic drinking water concentration ( μg˙•L^-1^). Period of samples collection not reported.	ICD10 C67			
			Low (0–40)		SMR_male_	–	3.14 (2.9–3.4)
			Medium (40–320)			–	4.0 (3.6–4.5)
			High (320–1,800)			–	4.7 (4.1–5.4)
			Low (0–40)		SMR_female_	–	1.0 (reference)
			Medium (40–320)			–	0.94 (0.84–1.1)
			High (320–1,800) [Arsenic measurements from many surveys, one dating 50 years prior to study publication but with arsenic levels showing high degree of consistency with a more recent survey with no exact date detailed.]			–	1.2 (1.04–1.4)
*†Su et al. 2011 [64] (Table Two)	BFD endemic area, Taiwan	Mortality 1979-2003	Median arsenic content of artesian well: 780 μg˙•L-1 (range: 350–1,140). [Period of samples collection not reported. Artesian wells in the region were dug in the 1920s but no longer used by mid-1970s. Results show a comparison of mortality in BFD endemic area with that of Taiwan.]	ICD9 188	SMR	785	5.3 (4.9–5.6)
†Aballay et al. 2012^11^[62] (Table Two)	123 districts in province of Cordoba, Argentina	Incidence 2004	Arsenic water samples from 3 aquifers: (1) Rjojan plain (concentration ranged 0–40 μg˙•L^-1^ - 23 wells), (2) Pampean mountains (0–320 μg˙•L^-1^- 114 wells) and (3) Chaco-Pampean plain (0–1,800 μg˙•L^-1^ - 301 wells). In 80 wells, arsenic was undetected.	N/A	RR_male_	–	13.8 (6.80–28.0)
					RR_female_	–	12.7 (2.51–63.9)
†Fernández et al. 2012 [55]	Antofagasta, Chile	Mortality 1983-2009	Arsenic drinking water concentration ranging 800–900 μg˙•L^-1^. [Arsenic levels based on the last 60 years and obtained from the local tap water company in Antofagasta. Results compares mortality rate in Antofagasta with the rest of Chile.]	ICD10 C67	RR_male_	–	5.3 (4.8–5.8)
					RR_female_	–	7.8 (7.0–8.7)
					RR_both_sex_	–	6.1 (5.7–6.6)

*Study included in meta-analyses.

†Recent study not included in the International Agency for Research on Cancer 2012 review (Monograph 100C [23]).

^1^ All ecological studies assessed arsenic exposure at the group-level.

^2^ICD = International Classification for Disease for cancer site abstracted which included, bladder and urothelial/transitional cell carcinoma of the bladder or kidney. Transitional cell carcinoma of the renal pelvis often share the same etiology as bladder cancer, and as such, have been treated as bladder within the meta-analyses as recommended by IARC [23]. N/A = not available.

^3^SMR, standardized mortality ratio.

^4^Age-standardized mortality rates per 100,000 using the 1976 world population as standard population and based on 899,811 person-years.

^5^All age-standardized mortality rates shown are significant at p < 0.001 based on trend test.

^6^ Regression coefficient showing an increase in age-adjusted mortality per 100,000 persons-years for every 0.1 ppm increase in arsenic level, adjusting for indices of industrialization and urbanization. Standard errors are in brackets. Bladder cancer was significantly correlated with average arsenic level in water.

^7^Incidence rate per 100,000, adjusted for age.

^8^County is the unit of analysis.

^9^RD, rate difference (per 100,000 person-years) for one unit increase in the predictor and associated standard error for exposure > 640 μg˙•L^-1^(SE). Results shown for transitional-cell carcinoma.

^10^Average annual age-adjusted (to U.S. 1970 standard population) death rates per 100,000 abstracted at the state level for each decade were used as standard rates to calculate county-specific SMRs.

^11^Incidence rate ratio estimates with arsenic as continuous.

^12^Used lung cancer mortality rates as surrogate to smoking - may result in an overestimation of risk where smoking has declined; an underestimation of risk where smoking has increased; and an over-adjusted model as lung cancer is also associated with arsenic exposure.

**Table 3:** Summary results from case–control studies reporting on arsenic exposure and the risk of bladder cancer

**Study [reference] (Table from original publication)**	**Study locale**	**Outcome**	**ICD**^**1**^	**Arsenic exposure assessment**	**Exposure [comments]**	**Cases: Controls**	**All participants**		**Never smokers**		**Ever smokers**		**Covariates assessed**
							**n**	**OR**^**2**^**, (95% CI)**	**n**	**OR, (95% CI)**	**n**	**OR, (95% CI)**	
Chen et al. 1986^3^[25] (Table Four)	4 neighbouring Blackfoot disease (BFD)-endemic areas, Taiwan	Mortality 1996-2000	N/A	Individual level ‘estimated’	Year of artesian water consumption:	69:368							age, sex, cigarette smoking, tea drinking habit, vegetarian habit, vegetable consumption frequency, fermented bean consumption frequency
					0 (referent)		17	1.0	–	–	–	–	
					1 – 20		19	1.27	–	–	–	–	
					20 – 40		10	1.68	–	–	–	–	
					≥ 40		23	4.10	–	–	–	–	
					[Median arsenic content of artesian wells and (range): 780 μg˙•L^-1^ (350 – 1,140). History of artesian well water noted.]								
Bates et al. 1995 [31] (Table Three)	Utah, USA	Incidence	N/A	Individual level ‘measured’	Cumulative dose index of arsenic (mg):	117:266							age, sex, smoking, exposure to chlorinated surface water, history of bladder infection, education, urbanization of the place of longest lifetime residence, and ever employed in high-risk occupation
		Diagnosis in a 1-year period around 1978			< 19 (referent)		14	1.0	10	1.0	4	1.0	
					19 to < 33		21	1.56 (0.8–3.2)	10	1.09 (0.4–3.1)	11	3.33 (1.0–10.8)	
					33 to < 53		17	0.95 (0.4–2.0)	7	0.68 (0.2–2.3)	10	1.93 (0.6–6.2)	
					≥ 53		19	1.41 (0.7–2.9)	4	0.53 (0.1–1.9)	15	3.32 (1.1–10.3)	
					[Arsenic water concentration ranged 0.5 - 160 μg˙•L and averaged 5 μg˙•L. Data on arsenic levels in public drinking water supplies were collected in 1978–79. Results are based on the 71 cases who had lived in study towns for at least half of their lives. Residential history and water source used in exposure assessment.]								
*Kurttio et al. 1999 [20] (Tables Six, Seven)	Areas in Finland with < 10% population with municipal drinking-water system	Incidence 1981-95	N/A	Individual level ‘measured’	Arsenic water concentration (μg˙•L^-1^):	61:275							age, sex, smoking
					< 0.1		23	1.0	8	1.0	8	1.0	
					1.1 -0.5		19	1.53 (0.75–3.09)	4	0.95 (0.25–3.64)	3	1.10 (0.19–6.24)	
					≥ 0.5		19	2.44 (1.11–5.37)	5	0.87 (0.25–3.02)	7	10.3 (1.16–92.6)	
					(log) continuous [Only subjects with drilled wells; median total arsenic concentration of 0.1 μg˙•L ; max.concentration of 64 μg˙•L and 1% exceeding 10 μg˙•L. Water sampled from wells used by the study population at least for 1967–80. Exposure in the 3rd-9th calendar year prior to cancer diagnosis. Residential history and drinking water consumption used in exposure assessment.]		61	1.37 (0.95–1.96)		–		–	
Chen et al. 2003 [91] (Table Two)	Southwestern Taiwan	Incidence 1996-99	ICD9 188	Individual level ‘estimated’	Cumulative arsenic exposure (mg˙•L^-1^•year):	49:224							age, sex, BMI, cumulative arsenic exposure, cigarette smoking, hair dye usage, education
					0 – 2		30	1.0	–	–	–	–	
					> 2 – 12		4	0.6 (-1.1–3.0)	–	–	–	–	
					> 12		10	1.86 (0.2–5.10)	–	–	–	–	
					[Arsenic concentration in artesian well water from survey of 83,656 wells between 1974–76. Questionnaires used to determine village in which subjects lived 30 years ago. Residential history and duration and; source of drinking water used in exposure assessment.]								
Steinmaus et al. 2003 [92] (Tables Three, Four)	6 counties in Nevada; 1 county in California, USA	Incidence 1994-2000	N/A	Individual level ‘estimated’	Cumulative exposure to arsenic in water (mg˙•L^-1^•year):	181:328							OR for all participants adjusted for age, gender, occupation, smoking history (<1 pack per day (ppd), ≥1 ppd, former smoker, never smoker), income, education and race
					< 6.4		153	1.0	23	1.0	130	1.0	
					6.4 – 82.8		9	1.63 (0.64–4.13)	3	2.65 (0.49–14.2)	6	1.06 (0.34–3.33)	
					> 82.8		19	1.40 (0.73–2.70)	3	0.50 (0.12–2.05)	13	2.25 (0.97–5.20)	
					[Arsenic concentration from 7,000 samples from community and domestic wells. Results for a 40 years lagged exposure; 88.4% of cases and 91.8% of controls being exposed to arsenic levels ranging from 0 to 19 μg˙•L, respectively. Residential history, source of drinking water and intake used in exposure assessment.]								
*Bates et al. 2004 [93] (Tables Two, Three)	Cordoba, Argentina	Incidence 1996-2000	N/A	Individual level ‘measured’	Arsenic water concentration (μg˙•L^-1^):	114:114							*mate con bombilla* consumption, education, and home tap-water consumption in all groups; and adjusted for the highest daily number of cigarettes subjects reported ever having smoked in the smoker group
					0–50		70	1.0	22	1.0	65	1.0	
					51–100		13	0.88 (0.3–2.3)	2	1.05 (0.2–6.9)	7	1.29 (0.3–5.0)	
					101–200		22	1.02 (0.5–2.3)	3	1.10 (0.2–6.3)	10	0.96 (0.3–3.0)	
					> 200		9	0.60 (0.2–1.7)	1	0.58 (0.1–6.2)	2	0.17 (0.0–1.0)	
					[Average arsenic concentration of 5 years of highest exposure during the period 6–40 years before interview. On average, cases and controls had 25.7 and 25.6 years of well-water consumption, respectively; also approximately 50% of all well years were derived from proxy-well data. Results shown for transitional cell bladder cancer.]								
Karagas et al. 2004 [94] (Table Two)	New Hampshire, USA	Incidence 1994-98	N/A	Individual level ‘measured’	Arsenic toenail concentration (μg˙•g^-1^):	383:641							age, sex, smoking status (ever/never)
					0.009–0.059		90	1.0	15	1.0	75	1.0	
					0.060–0.086		119	1.37 (0.96–1.96)	20	0.85 (0.38–1.91)	99	1.53 (1.02–2.29)	
					0.087–0.126		88	1.08 (0.74–1.58)	22	1.18 (0.53–2.66)	66	1.02 (0.66–1.56)	
					0.127–0.193		48	1.04 (0.66–1.63)	11	1.10 (0.42–2.90)	37	1.00 (0.60–1.67)	
					0.194–0.277		2	1.33 (0.71–2.49)	3	0.49 (0.12–2.05)	18	1.78 (0.86–3.67)	
					0.278–0.330		3	0.41 (0.11–1.50)	0	–	3	0.50 (0.13–1.88)	
					0.331–2.484		14	1.36 (0.63–2.90)	0	–	14	2.17 (0.92–5.11)	
					[Levels of arsenic in toenails reflect exposures occurring between 9–15 months prior to sample collection. On average cases and controls had 16.5 and 17.2 years exposure to their water system. Results shown for transitional cell bladder cancer.]								
Michaud et al. 2004 [95] (Table Two)	Southwestern Finland	Incidence 1985-99	ICD9 188, 233.7	Individual level ‘measured’	Arsenic toenail concentration (μg˙•g^-1^):	280:293							age, toenail collection date, intervention group, number of cigarettes per day, and number of years smoking
					< 0.105		–	–	–	–	136	1.0	
					0.105–0.160		–	–	–	–	73	1.10 (0.73–1.64)	
					0.161–0.259		–	–	–	–	37	0.93 (0.56–1.54)	
					0.260–0.399		–	–	–	–	20	1.38 (0.68–2.80)	
					> 0.399		–	–	–	–	14	1.14 (0.52–2.51)	
† Pu et al. 2007 [51] (Tables Four, Five)	Taiwan	Incidence 2002-04	N/A	Individual level ‘measured’	Arsenic urine concentration (μg˙•g^-1^ creatine):	177:313							OR (all participants): age, sex, education, parents’ ethnicity, alcohol drinking, pesticides use
					≤ 15.4		24	1.0	–	–	–	–	
					15.5–26.4		44	1.9 (1.1–3.4)	–	–	–	–	
					>26.4		109	5.3 (3.1–9.0)	–	–	–	–	
					≤ 20.3		–	–	17	1.0	21	1.0	OR (never/ever smokers): age, sex
					≥ 20.3		–	–	68	4.4 (2.3–8.5)	61	8.2 (3.8–17.8)	
					[Smokers include current and former smokers. Non-smokers with ≤ 20.3 (μg˙•g^-1^ creatine) was used as referent category.]								
*†Meliker et al. 2010 [87] (Table Three)	11 counties of Southeastern Michigan, USA	Incidence 2000-04	N/A	Individual level ‘measured’	Arsenic water concentration (μg˙•L^-1^):	411:566							age, sex, race, smoking history, education, history of employment in high risk occupation, family history of bladder cancer
					< 1		187	1.0	–	–	–	–	
					1–10		182	0.84 (0.63–1.12)	–	–	–	–	
					> 10		38	1.10 (0.65–1.86)	–	–	–	–	
					[Arsenic water concentrations obtained from: 6,050 private untreated wells sampled between 1993–2002; 371 well water measurements from participants’ current residence and; 1,675 measurements from public well water supplies collected between 1983–2004, which were used to estimate arsenic concentrations at past residences.]								
*†Steinmaus et al. 2013 [67] (Table Two)	Region I and II, northern Chile	Incidence 2007-10	N/A	Individual level ‘estimated’	Arsenic water concentration (μg˙•L^-1^):	306:640							no covariates assessed, although subjects were frequency matched on age, sex
					0–59		23	1.0	–	–	–	–	
					60–199		27	0.84 (0.46–1.52)	–	–	–	–	
					200–799		60	2.50 (1.48–4.22)	–	–	–	–	
					> 800		122	4.44 (2.75–7.15)	–	–	–	–	
					[Each city/town of residence in which each subject lived was linked to a water arsenic measurement for that city/town so that an arsenic concentration could be assigned to each year of each subject’s life. Study also present OR in relation to various metrics of arsenic exposure such as lifetime and cumulative average exposure and; lifetime and cumulative intake. Residential history used in exposure assessment.]								

*Study included in meta-analyses.

†Recent study not included in the International Agency for Research on Cancer 2012 review (Monograph 100C [23]).

^1^ICD = International Classification of Disease. N/A = not available.

^2^OR = Odds ratios.

^3^OR crude = 1.0, 1.17, 1.60, 3.90 for corresponding years of exposure shown in table.

**Table 4:** Summary results from cohort studies reporting on arsenic exposure and the risk of bladder cancer

**Study [reference] (Table from original publication)**	**Study locale**	**Outcome**	**ICD1**	**Arsenic exposure assessment**	**Exposure [comments]**	**Outcome measure**	**Cohort size**	**Cases**	**Risk estimate (95% CI)**	**Covariates assessed**
Chen et al. 1988 [70] (Table Six)	4 neighbouring townships from Blackfoot disease (BFD) endemic area, Taiwan	Morality 1968-83	N/A	Group level	Median arsenic content of artesian well and (range): 0.78 ppm (0.35–1.14); in shallow well: 0.04 (0.00-0.30). General population used as reference. 95% CI obtained from IARC 2012 review [23].	SMR	871	15	38.8 (21.7–64.0)	
Chiou et al. 1995 [32] (Table Four)	4 neighbouring townships from BFD endemic area, Taiwan	Incidence 1988 (Follow-up period ranged 0.05 to 7.7 years)	N/A	Individual level ‘estimated’	Cumulative arsenic exposure (mg˙•L^-1^˙•year):	RR	2,556	29		age, sex, cigarette smoking
					0				1.0	
					0.1–19.9				1.57 (0.44–5.55)	
					> 20				3.58 (1.05–12.19)	
					unknown				1.25 (0.38–4.12)	
					[Median arsenic content of artesian well and (range): 0.78 ppm (0.35–1.14); in shallow well: 0.04 (0.00-0.30). Histories of residential address and duration of drinking well water used to derive cumulative exposure.]					
*Tsuda et al.^2^ 1995 [34] (Table Three)	Niigata, Japan	Mortality 1959-92 (Recruitment in 1959, followed until 1992)	Transitional cell carcinoma	Individual level ‘measured’	Arsenic water concentration (μg˙•L^-1^):	SMR	443			age, smoking habits
					< 50			254	0.00 (0–12.50)	
					50 – 990			76	0.00 (0–47.05)	
			ICD9 188, 189 ICDO histology N/A		≥ 1,000			113	31.18 (8.62–91.75)	
					[Arsenic-polluted area. Exposure to be between 1955-59. All 34 wells in the area were sampled and arsenic concentration ranged from non detectable to 3,000 μg˙•L^-1^).]					
Lewis et al. 1999^3^[40] (Table Four)	Millard County in Utah, USA	Mortality (Recruitment 1900–1945)	N/A	Group level	Cumulative arsenic exposure derived from: low exposure (< 1000 ppb-year); medium (1,000-4,999 ppb-year); high (≥ 5,000 ppb-year):		4,058			Individual data on cofactors not available. However, the cohort was assembled from historical membership records of the Church of Jesus Christ of Latter-day Saints (Mormons) which prohibits tobacco use and the consumption of alcohol and caffeine.
						SMR_male_		–	0.42 (0.08–1.22)	
					< 1,000 ppb•year	SMR_female_		–	0.81 (0.10–2.93)	
					≥ 5,000 ppb•year	SMR_male_		–	0.4	
					[Residential history combined with local water records used to assess exposure. High variability in exposure estimates in each community with median arsenic concentrations ranging from 14 to 166 ppb. Records of arsenic measurements dating back to 1964.]	SMR_female_		–	1.18	
						SMR_male_		–	0.95	
						SMR_female_		–	1.10	
*Chiou et al. 2001^3^[33] (Table Five)	18 villages in four townships in Lanyang Basin, North-eastern Taiwan	Incidence 1991-1994 (Follow-up period from time of enrollment to Dec.1996)	Urinary organs	Individual level ‘estimated’	Arsenic water concentration (μg˙•L^-1^):	RR	8,102			age, sex, cigarette smoking, duration of well water drinking
					0–10.0	Urinary organs		3	1.0	
			ICD9 188, 189							
					10.1–50.0			3	1.5 (0.3–8.0)	
					50.1–100.0			2	2.2 (0.4–13.7)	
			Transitional cell carcinoma		> 100.0			7	4.8 (1.2–19.4)	
					Arsenic water concentration (μg˙•L^-1^);	RR Transitional cell carcinoma				
					0–10.0			1	1.0	
			ICDO1 8120.2, 8120.3, 8130.3		10.1–50.0			1	1.9 (0.1–32.5)	
					50.1–100.0			2	8.2 (0.7–99.1)	
					> 100.0			6	15.3 (1.7–139.9)	
					[Arsenic levels in shallow well ranging from < 0.15 to 3,590 μg˙•L^-1^ and collected from 3,901 well water samples between 1991–94.]					
† Baastrup et al. 2008 [96] (Table Three)	23 municipalities in Copenhagen & Asrhus areas, Dannemark	Incidence 1993-1997 (Follow-up from enrollment until date of first cancer diagnosis, emigration, death, or Aug. 2003)	N/A	Individual level ‘estimated’	Cumulated arsenic exposure (5 mg˙):	IRR	56,378	214	1.0 (0.98–1.04)	smoking status, smoking duration, smoking intensity, education, occupation
					Time-weighted average exposure (μg˙•L^-1^):	IRR		214	1.01 (0.93–1.11)	
					[Average arsenic exposure from 0.05 to 25.3 μg˙•L^-1^, with mean of 1.2 μg˙•L^-1^. Average arsenic concentrations obtained from 4,954 samples from 2,487 water utilities collected, 1987–2004, with most samples dating 2002–04. Residential history 1970–2003.]					
*†Huang et al. 2008 [53] (Table Two)	3 villages in Putai Township, in BFD endemic area of southern Taiwan	Incidence 1989 (Average follow-up period of 12 years)	Urothelial carcinoma	Individual level ‘estimated’	Arsenic water concentration (μg˙•L^-1^):	RR	1,078			age, sex, cigarette smoking, education
					0–400			1	1.0	
			ICDO3 M-codes 8120/3, 8230/3		401–700			14	5.2 (0.7–39.8)	
					710–900			9	6.7 (0.8–53.4)	
					≥ 900			7	6.5 (0.8–53.1)	
					Cumulative arsenic exposure (mg˙•L^-1^•year):	RR				
					0			0	–	
					0.1–11.9			2	1.0	
					12.0–19.9			9	4.6 (1.0–21.8)	
					≥ 20.0			20	7.9 (1.7–37.9)	
					[Period of arsenic water samples collection not reported. Participants used artesian well water more > 30 years when recruited. Information from interview included history of well-water consumption, residential history, lifestyle factors].					
*†Chen et al. 2010^5^[60] (Tables One, Two)	Taiwan	Incidence 1991-1994 (Average follow-up period of 11.6 years)	Urothelial carcinoma	Individual level ‘measured’	Arsenic water concentration (μg˙•L^-1^):	RR	8,086			age, sex, cigarette smoking status, education, alcohol consumption at enrolment, and whether subject started drinking well water from birth
			ICDO histology		< 10	Urothelial carcinoma		3	1.0	
			N/A		10–49.9			6	1.85 (0.45–7.61)	
			Urinary organs		50–99.9			3	2.19 (0.43–11.1)	
			ICD9 188, 189, 189.1-189.9		100–299.9			7	5.50 (1.39–21.8)	
					≥ 300			10	10.8 (2.90–40.3)	
					unknown			7	4.34 (1.06–17.7)	
					Cumulative arsenic exposure (μg˙•L^-1^•year):					
					< 400	RR		6	1.0	
					400– < 1,000	Urinary organs		3	1.16 (0.29–4.64)	
					1,000– < 5,000			12	2.44 (0.91–6.50)	
					5,000– < 10,000			5	3.88 (1.18–12.7)	
					≥ 10,000			11	7.55 (2.79–20.4)	
					Unknown			8	2.90 (1.01–8.37)	
					[Arsenic concentration ranged < 0.15 to > 3,000 μg˙•L^-1^ and was estimated using 3,901 water samples from residence of participants at time of interview. Other measures of arsenic exposure included, duration of exposure, age starting/ending drinking well water, and cumulative exposure.]					
*†Chung et al. 2013^6^[65] (Table One)	3 villages in Putai Township, in BFD endemic area of southern Taiwan	Mortality 1996-2010 (Average follow-up period of 17.8 years)	ICD9 188	SMR based analyses:	Median arsenic content of artesian well (range: 700–930 μg˙•L^-1^) measured in the early 1960s.	SMR_male_	1,563	24	2.9 (27.5–63.8)	SMR adjusted for age
						SMR_female_		19	59.4 (35.7–92.7)	
				Group level						
					[Used age-adjusted mortality rate in Taiwan as standard rates.]					
				HR based analyses: Individual level ‘estimated’	Average arsenic concentration in artesian well (μg˙•L^-1^):	HR				HR adjusted for age, gender, education, smoking habits
					< 50			1	1.0	
					50–710			15	4.35 (0.56–33.52)	
					> 710			22	7.22 (0.95–55.04)	
					[Duration of drinking artesian well water and history of residential address obtained from questionnaires. Authors found a significant association with duration of well water drinking.]					

*Study included in meta-analyses.

†Recent study not included in the International Agency for Research on Cancer 2012 review (Monograph 100C [23]).

^1^ICD = International Classification of Disease. ICD for cancer site abstracted which included bladder and urothelial/transitional cell carcinoma of the bladder or kidney. Transitional cell carcinoma of the renal pelvis often share the same etiology as bladder cancer, and as such, have been treated as bladder within the meta-analyses as recommended by IARC [23]. N/A = Not available.

^2^Cases = number of persons exposed between 1955-1959.

^3^95% Confidence intervals not available for data at low and high exposure.

^4^Results for transitional cell carcinoma were included in the meta-analysis.

^5^Results for urothelial carcinoma were included in the meta-analysis.

^6^Results from SMR were included in the meta-analyses.

**Table 5:** Summary results from ecological studies reporting on arsenic exposure and kidney cancer

**Study [reference] (Table from original publication)**	**Study locale**	**Outcome**	**Exposure**^**1**^**[comments]**	**ICD**^**2**^	**Outcome measure**	**Cases**	**Risk estimate (95% CI)**
Chen et al. 1985^3^[24] (Table One)	84 villages from 4 neighbouring townships on SW coast, Taiwan	Mortality 1968-82	Median arsenic content of artesian well and (range): 780 μg˙•L^-1^ (350–1,140); in shallow well: 40 (0.0–300). Period of samples collection not reported.	ICD 189	SMR_male_	42	7.72 (5.37–10.1)
			[Comparison of mortality rate in Blackfoot disease (BFD) with those of the general population.]		SMR_female_	62	11.2 (8.38–14.0)
*Chen et al. 1988^4^[26] (Table One)	BFD endemic area, Taiwan	Mortality 1973-86	Arsenic well water concentration (μg˙•L^-1^). Period of samples collection not reported.	ICD 189			
			General population		ASMR_male_	–	1.1
						–	5.4
			< 300			–	13.1
			300-590			–	21.6
			≥ 600				
			General population		ASMR_female_	–	0.9
						–	3.6
			< 300			–	12.5
			300-590			–	33.3
			≥ 600				
			[Comparison of mortality rate in BFD with those of the general population.]				
*Wu et al. 1989^5^[27] (Table Three)	BFD endemic area, Taiwan (42 villages)	Mortality 1973-86	Arsenic well water concentration (μg˙•L^-1^) based on well water samples collected between 1964–66.	ICD8 189			
			< 300		ASMR_male_	9	8.42
						11	18.9
			300–590			6	25.3
			≥ 600				
			< 300		ASMR_female_	4	3.42
						13	19.4
			300–590			16	58.0
			≥ 600				
Chen and Wang 1990^6^[28] (Table Four)	314 precincts & townships in Taiwan, including 4 from BFD endemic area	Mortality 1972-83	Average arsenic levels in water samples of all 314 geographical units. 73.9% had < 5% of wells with > 50 μg˙•L^-1^ ; 14.7% had 5-14%; 11.5% had ≥ 15%. Well water samples collected between 1974–76.	ICD 189			
			All precincts & townships		ASMR_male_	–	1.1 (0.2)
					ASMR_female_	–	1.7 (0.2)
			Southwestern townships		ASMR_male_	–	1.2 (0.2)
					ASMR_female_	–	1.7 (0.3)
Guo et al. 1997^7^[37] (Table Two)	243 townships in Taiwan	Incidence 1980-87	Arsenic well water concentration ranging from < 50 to > 640 μg˙•L-^1^.	ICD 189.0, 189.1	RDmale	–	0.03 (0.02)
			Estimate presented measured at > 640 μg˙•L^-1^. [Arsenic measurements from a National survey of 83,656 wells in 243 townships, collected mostly between 1974–76.]		RDfemale	–	0.14 (0.013)
Rivara et al.1997 [38] (Table Four)	Chile	Mortality 1950-92	Annual average arsenic concentration in drinking water for Antofagasta (Region II of Chile) ranging between 40 to 860 μg˙•L^-1^. Data from historical records from 1950–1992.	ICD 189	RR	–	3.8 (3.1–4.7)
			[Comparison of mortality rate in Region II (exposed) populations vs Region VIII (control population.]				
Smith et al. 1998 [39]	Chile	Mortality 1989-93	Region II of Northern Chile with population weighted average arsenic concentration in drinking water up to 569 μg˙•L^-1^ compared with the rest of Chile; exposure generally < 10 μg˙•L^-1^.	N/A	SMR_male_	39	1.6 (1.1–2.1)
			[Arsenic measurements from 1950–94.]		SMR_female_	34	2.7 (1.9–3.8)
Hinwood et al. 1999 [88] (Table Two)	22 areas in Victoria, Australia	Incidence 1982-91	Median water arsenic concentration ranging 13 μg˙•L^-1^ to 1,077 μg˙•L^-1^. [Selected areas were those where samples with soil and/or water arsenic concentration were generally in excess of 10 μg˙•L^-1^. Period for samples collection is not available.]	ICD 189.0, 189.9	SIR	134	1.16 (0.98–1.37)
*Tsai et al. 1999 [41] (Tables Two, Three)	4 townships from BFD endemic area in SW coast, Taiwan	Mortality 1971-94	Median arsenic content of artesian well: 780 μg˙•L^-1^ (range: 350–1,140).	ICD 189	SMR_local-male_	94	6.76 (5.46–8.27)
					SMR_national-male_	94	6.80 (5.49–8.32)
			Period of samples collection not reported. Authors state that artesian wells were no longer used by the mid-1970s.		SMR_local-female_	128	8.89 (7.42–10.6)
			[Comparison of mortality in BFD endemic area with that of a local reference population (Chiayi-Tainan county) and that of Taiwan as a whole.]		SMR_national-female_	128	10.5 (8.75–12.5)
*†Meliker et al. 2007 [90] (Table Two)	6 counties, Southeastern Michigan, USA	Mortality 1979-97	Population weighted median arsenic concentration in water of 7.58 μg˙•L^-1^, with a range between 10–100 μg˙•L^-1^. Data from 9,251 well water samples collected between 1983–2002.	ICD9 189	SMR_male_	325	1.06 (0.91–1.22)
					SMR_female_	194	1.00 (0.82–1.20)
†Yuan et al. 2010 [61] (Tables Two, Three)	Region II and V, Chile	Mortality 1950-2000	Northern Chile (Region II) with population weighted average arsenic concentration in drinking water up to 569 μg˙•L^-1^ vs Region V with exposure close to 1 μg˙•L^-1^. Between 1958-70, arsenic concentration in water supply of Antofagasta and nearby Mejillones (Region II) averaged 870 μg˙•L^-1^ and declined in 1970s when treatment plants were installed.	ICD9 189; ICD10 C64-C66, C68	Men and women aged 30+ years		
					RR_male-1950–54_	4	0.69 (0.23–2.02)
					RR_male-1955–59_	9	1.43 (0.66–3.10)
					RR_male-1960–64_	7	0.91 (0.40–2.08)
					RR_male-1965–69_	12	2.51 (1.22–5.17)
					RR_male1970–74_	15	1.45 (0.81–2.60)
					RR_male1975–80_	19	2.13 (1.24–3.68)
					RR_male1981–85_	39	3.37 (2.21–5.11)
					RR_male1986–90_	63	2.81 (2.05–3.85)
					RR_male1991–95_	50	1.78 (1.28–2.47)
					RR_male1996–00_	66	1.61 (1.21–2.14)
					RR_female-1950–54_	2	1.27 (0.27–6.00)
					RR_female-1955–59_	2	0.30 (0.07–1.25)
					RR_female-1960–64_	7	1.66 (0.71–3.91)
					RR_female-1965–69_	3	0.76 (0.23–2.57)
					RR_female1970–74_	13	3.70 (1.81–7.56)
					RR_female1975–80_	9	1.71 (0.80–3.65)
					RR_female1981–85_	25	2.89 (1.77–4.72)
					RR_female1986–90_	41	3.23 (2.18–4.78)
					RR_female1991–95_	49	4.37 (2.98–6.41)
					RR_female1996–00_	47	2.32 (1.64–3.28)
					Young adults aged 30-39 years, born during and just before high-exposure period; and for ages 40+, born before 1950 with no early life exposure.		
					SMR_male_30-49 years_	4	5.63 (1.52–14.4)
					SMR_male_40 years+_	103	2.68 (2.19–3.26)
					SMR^female_30-49 years^	4	9.52 (2.56–24.4)
					SMR_female_40 years+_	84	3.91 (3.12–4.84)
					SMR_total_30-49 years_	8	7.08 (3.05–14.0)
					SMR_total_40 years+_	187	3.12 (2.69–3.61)

*Study included in meta-analyses.

†Recent study not included in the International Agency for Research on Cancer 2012 review (Monograph 100C [23]).

^1^All ecological studies assessed arsenic exposure at the group-level.

^2^ICD = International Classification of Disease. N/A = not available.

^3^SMR, standardized mortality ratio.

^4^Age-standardized mortality rates per 100,000 using the 1976 world population as standard population and based on 899,811 person-years.

^5^All age-standardardized mortality rates shown are significant at p < 0.001 based on trend test.

^6^Regression coefficient showing an increase in age-adjusted mortality per 100,000 persons-years for every 0.1 ppm increase in arsenic level, adjusting for indices of industrialization and urbanization. Standard errors are in brackets. Kidney cancer was significantly correlated with average arsenic level in water.

^7^RD, rate difference (per 100,000 person-years) for one unit increase in the predictor and associated standard error for exposure > 640 μg˙•L^-1^(SE).

**Table 6:** Summary results from cohort studies reporting on arsenic exposure and risk of kidney cancer

**Study [reference] (Table from original publication)**	**Study locale**	**Outcome**	**ICD**^**1**^	**Arsenic exposure assessment**	**Exposure [comments]**	**Outcome measure**	**Cohort size**	**Cases**	**Risk estimate (95% CI)**	**Covariates assessed**
Chen et al. 1988 [70] (Table Six)	4 neighbouring townships from Blackfoot disease (BFD) endemic area, Taiwan	Morality 1968-83	N/A	Group level	Median arsenic content of artesian well and (range): 0.78 ppm (0.35–1.14); in shallow well: 0.04 (0.00-0.30). General population used as reference. 95% CI obtained from IARC 2012 review [23].	SMR	871	3	19.5 (4.0–57.0)	
Lewis et al. 1999^2^[40] (Table Four)	Millard County in Utah, USA	Mortality (Recruitment 1900–1945)	N/A	Group level	Cumulative arsenic exposure derived from: low exposure (< 1000 ppb-year); medium (1,000-4,999 ppb-year); high (≥ 5,000 ppb-year):	SMR_male_	4,058	–	1.75 (0.80–3.32)	Individual data on cofactors not available. However, the cohort was assembled from historical membership records of the Church of Jesus Christ of Latter-day Saints (Mormons) which prohibits tobacco use and the consumption of alcohol and caffeine.
						SMR_female_		–	1.60 (0.44–4.11)	
					< 1,000 ppb•year	SMR_male_		–	2.5	
						SMR_female_		–	2.4	
					1,000 - 4,999 ppb•year	SMR_male_		–	1.1	
						SMR_female_		–	1.3	
					≥ 5,000 ppb•year [Residential history combined with local water records used to assess exposure. High variability in exposure estimates in each community with median arsenic concentrations ranging from 14 to 166 ppb. Records of arsenic measurements dating back to 1964.]	SMR_male_		–	1.4	
						SMR_female_		–	1.1	
†Baastrup et al. 2008 [96] (Table Three)	23 municipalities in Copenhagen & Asrhus areas, Dannemark	Incidence 1993-1997 (Follow-up from enrollment until date of first cancer diagnosis, emigration, death, or Aug. 2003)	N/A	Individual level ‘estimated’	Cumulated arsenic exposure (5 mg˙):	IRR	56,378	53	0.94 (0.84–1.06)	smoking status, smoking duration, smoking intensity, education, occupation
					Time-weighted average exposure (μg˙•L^-1^):	IRR		53	0.89 (0.65–1.21)	
					[Average arsenic exposure from 0.05 to 25.3 μg˙•L^-1^, with mean of 1.2 μg˙•L^-1^. Average arsenic concentrations obtained from 4,954 samples from 2,487 water utilities collected, 1987–2004, with most samples dating 2002–04. Residential history 1970–2003.]					

†Recent study not included in the International Agency for Research on Cancer 2012 review (Monograph 100C [23]).

^1^ICD = International Classification of Disease. N/A = not available.

^2^95% Confidence intervals not available for data at low, medium and high exposure.

### Data analysis

Epidemiologic data from studies which explicitly provided point estimates of As levels in drinking water were used in a meta-analysis to examine the association between cancer outcomes and As exposure over a broader and more continuous range of As than previously available (Tables 2, 3, 4, 5, 6, studies with an asterisk). Studies using cumulative exposure to As in drinking water, years of artesian well water consumption or As toenail/urine concentrations were not included in the meta-analyses. Risk estimates from studies reporting on bladder cancer mortality (10 studies) were analysed separately from those reporting on incidence (7 studies). With regards to kidney cancer, only risk estimates for mortality could be analysed (2 studies) as there were insufficient studies reporting on kidney cancer incidence.

Combined risk estimates from studies reporting on standardized mortality ratios (SMR) were modeled using a least squares linear regression model for the logged SMRs; studies reporting mortality rates or relative risk (RR – incidence data only) were analyzed with a Generalized Linear Model having a Gamma-distributed response and a log link function, a combination well suited to analyses with highly variable risk estimates [97]. Risk estimates were modeled as a function of logged As and a categorical variable with a level for each study. The latter accounted for possible variations in baseline risk between studies due to differing methodological designs, study quality, populations, etc., and was assumed to be a fixed effect (herein, referred to as Model I, see Boreinsteign et al. [98]). The robustness/sensitivity of the predicted risk estimates obtained with the fixed effects As-risk models was assessed with bootstrap randomizations (10,000 permutations) which estimated the effect size at 10, 50 and 150 μg/L of As in drinking water (herein, referred to as Model II, see Efron and Tibshirani [99]). A random effects assumption was also examined; however, the small number of studies entering each model precluded a stable estimation of the variance components. Meta-analyses (Model I and II) modeling SMR and RR were only performed for bladder cancer due to the limited number of studies reporting on kidney cancer. Inference of risk at 10, 50 and 150 μg/L of As in drinking water and based on Model I, was only possible for bladder cancer incidence for which a reliable referent population and sufficient number of studies were available. Finally, the effect of sex and smoking on cancer risk was examined; however, analyses could not be completed due to insufficient degrees of freedom. Six of the 7 studies included in the meta-analysis of the RR had been adjusted for tobacco smoking in the original publication – an important risk factor in the development of urinary tract cancers and a possible effect modifier in the cancer-As relationship [51,86,100]. Only one of the 8 studies included in the analyses of the SMR adjusted for smoking [34], as these were generally ecological studies with no individual-level information on smoking. A list of covariates assesses in the original publication appear on Tables 3, 4, 6. Analyses were performed using R 2.13.0 [101].

## Results

### Study characteristics

**Figure 1:**
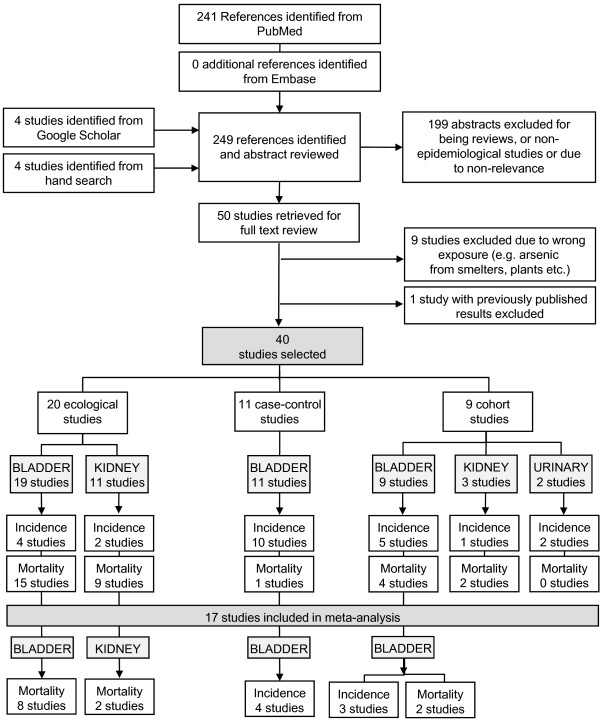
Study selection process. Note that several studies report on more than one cancer site.

**Figure 2:**
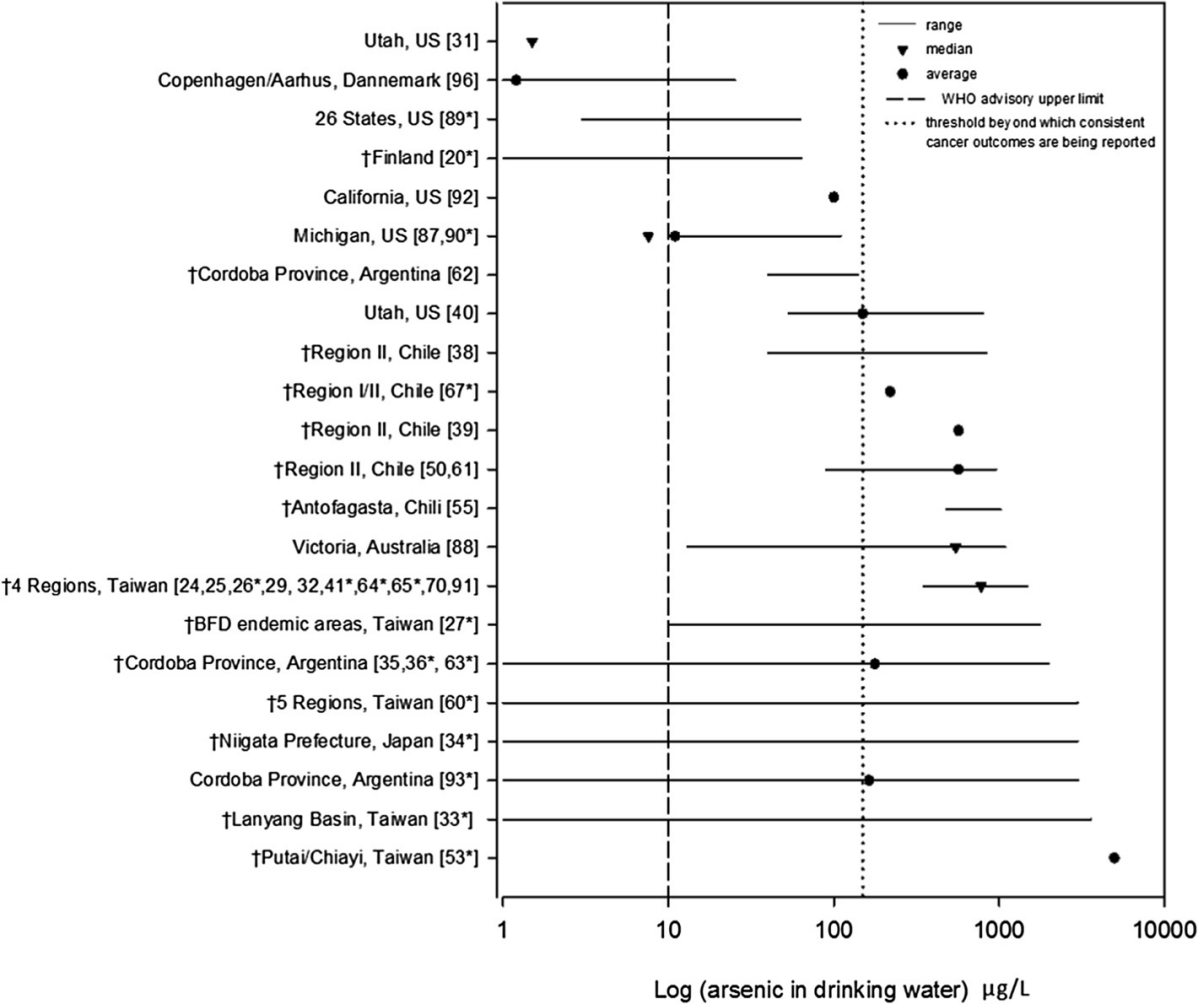
**Arsenic concentrations from studies reporting on urinary tract cancers outcomes and arsenic exposure in drinking water.** † indicates studies reporting significant associations and square brackets indicates citation number. Studies included in the meta-analysis are shown with an asterisk (*). Of the 40 studies reviewed, 3 used biomarkers to measure As exposure [51,94,95] and 2 failed to provide a specific measure of As-concentration [28,37].

The search resulted in the review of 249 abstracts, with 50 studies being retained for full text review (Figure 1). In total, forty studies met the inclusion criteria (principally, As in drinking water, toenail or urine as exposure measure and urinary tract cancer as outcome of interest) as listed in Table 1. Of these, 20 were ecological, 11 were case–control and 9 were cohort epidemiological studies. Thirty-seven of the 40 studies reported on bladder cancer outcomes and of these, 13 also reported on kidney cancer outcomes. One study focused exclusively on kidney cancer mortality [61]. Seventeen studies qualified for inclusion in the meta-analysis, 7 reporting on bladder cancer incidence and 10 on bladder cancer mortality. Two studies also reported on kidney cancer mortality, which was analysed independently from bladder cancer outcomes. Metrics of exposure included: As in well drinking water (median, average or range), cumulative As exposure, years of artesian well water consumption and As in toenails or urine. When measured in drinking water, exposure covered a broad spectrum of As concentrations, ranging from the study-specific detection limit to over 3,500 μg/L and with most study areas showing levels exceeding the WHO advisory limit (Figure 2). Adverse cancer outcomes were reported over the entire range of concentrations, although more consistently in regions where exposure levels were high, typically above 150 ug/L (Figure 2).

### Quality assessment

The quality of the studies was variable. For examples, all ecological studies assessed As exposure using group level (median or average) or ecologic measurements of drinking water (well or tap water), whereas all case–control and most cohort studies (7 of 9 studies) assessed As exposure using either a direct measure of As in tap/well water or body burden (e.g. urine or toenail As concentrations) or an individual level measure estimated from a range of metrics, including the reconstruction of past exposures based on residential history, knowledge of water source and duration of exposure to As contaminated well drinking water (see Table 2, 3, 4, 5, 6, As exposure assessment). Fifteen ecological studies and one cohort study stratified the analysis by gender (Tables 2, 4, 5, 6). With the exception of one study [70], all case–control and cohort studies included in this review accounted for tobacco smoking and one ecological study used lung cancer mortality rates as surrogate to smoking [63].

### Arsenic exposure and bladder cancer

#### Ecological studies

Fifteen of the 20 ecological studies reviewed reported on bladder cancer mortality (Table 2). These studies provided consistent evidence for an increased risk of death from bladder cancer with exposure to As in drinking water. There were two exceptions, however, they focused only upon low exposures (< 60 μg/L As in water; [89,90]). Risk estimates amongst males and females were comparable, with the exception of those reported by Chen et al. [24] which showed a near doubling of risk in females on the southwest coast of Taiwan (Table 2). Chen [26] was also first to describe a dose–response relationship between well water As and rates of mortality from bladder cancer. In accordance with the three levels of As exposure examined (< 300; 300 – 590; > 600 μg/L As), age-adjusted cancer mortality rates per 100,000 were as follows: 15.7, 37.8, 89.1 per 100, 000 males and 16.7, 35.1, 91.5 per 100,000 females. While these findings profiled the highly exposed populations of Taiwan, increased mortality from bladder cancer due to As exposure in drinking water was also observed in Argentina [35,36,62,63] and Chile [38,39,55]. For example, compared to un-contaminated areas, males and females from the highly contaminated Region II of Chile, experienced mortality rates due to bladder cancer, 6.0 and 8.2 times greater, respectively [39]. Within the same region, Rivara et al. [38] reported on mortality rates of an order of magnitude higher (sex combined) relative to those observed in the rest of Chile. Findings from the 4 ecological studies reporting on bladder cancer incidence were generally consistent with those of studies based on mortality, providing evidence for an association between bladder cancer and exposure to As in drinking water. The exception was a study by Hinwood et al. [88] which was limited by low power and exposure misclassification.

#### Case–control studies

Ten of the 11 case–control studies reviewed reported on bladder cancer incidence [20,31,51,67,87,91-95]; one reported on mortality ([25]; Table 3). Four studies observed a significant As-related increase in bladder cancer incidence; one study observed an increased risk of death with increasing years of artesian well water consumption in Blackfoot disease endemic areas of Taiwan ([25]; Table 3). Two of these studies assessed As exposure from As in tap/well water, one from urine, one from cumulated exposure and one from years of artesian well water consumption. Three of the five studies reporting a significant association, also provided risk estimates by smoking status [20,31,51]. Two studies failed to find an effect among non-smokers [20,31]; one study reported a risk of about half the magnitude of that observed among smokers (never smokers: 4.4 [2.3 – 8.5] vs smokers: 8.2 [3.8 – 17.8]; Table 3) [51]. Regardless of the type of metric used to measure exposure (i.e. cumulative dose index, As in drinking water, body burden etc.), the risk of developing bladder cancer as a result of exposure to As, was consistently higher among smokers.

#### Cohort studies

Five of the 9 cohort studies reviewed reported on bladder cancer incidence [32,33,53,60,96]; four reported on mortality (34,40,65,70]; Table 4). Seven of the 9 cohort studies showed an association between exposure to As contaminated drinking water and either bladder cancer incidence (4 studies, [32,33,53,60]) or mortality (3 studies, [34,65,70]). The work of both Chiou et al. [33] and Chen et al. [60] provided significant evidence for a dose–response relationship over a broad range of As exposure, from < 10 μg/L to ≥ 300 μg/L. Chen et al. [60] report relative risk estimates for bladder cancer increasing from 1.9, 2.2, 5.5 and 10.8 for exposure to As ranging from < 10, 10 – 49.9, 50 – 99.9, 100 – 299.9 and ≥ 300 μg/L, respectively. Consistent with these findings, Chiou et al. [33] report risks of similar magnitude, increasing from 1.9, 8.2, and 15.3 for exposure to As ranging from 10 – 50 μg/L, 50.1 – 100 μg/L and > 100 μg/L, respectively. The largest cohort study involving 56,378 cases failed to provide evidence of an association [96]. However, average exposure ranged of 0.05 and 25.3 μg/L and mean exposure level was 1.2 μg/L, with the authors indicating that only a small proportion of subjects were exposed to drinking-water containing As at > 2 μg/L. Eight of the 9 cohort studies retained in this review adjusted for the effect of tobacco smoking [32-34,40,53,60,65,96].

### As exposure and kidney cancer

#### Ecological studies

Nine of the 20 ecological studies reviewed reported on kidney cancer mortality (Table 5). Eight of these studies provided evidence for an increased risk of death from kidney cancer with exposure to As in drinking water [24,26-28,38,39,41,61]; one study found no association [90]. At high levels of As exposure risk estimates were generally higher amongst females. Chen [26] was again, first to describe a dose–response relationship between well water As and rates of mortality from kidney cancer, reporting age-standardized rates increasing from: 5.4, 13.1, 21.6 per 100, 000 males and 3.6, 12.5, 33.3 per 100,000 females, with exposure to < 300, 300 – 590, and > 600 μg/L As, respectively (Table 5). Two ecological studies reported on kidney cancer incidence [37,88] and one of these provided evidence for an association between kidney cancer and exposure to As in drinking water [37].

#### Case–control studies

None of the 11 case–control studies identified in this review reported on kidney cancer.

#### Cohort studies

One of the 9 cohort studies reported on kidney cancer incidence [96]; two reported on mortality [40,70] (Table 6). Of these 3 studies, one study showed a statistically significant increase in mortality with exposure to As contaminated drinking water [70]; the others reported a non significant increased risk in mortality [40] or incidence [96]. None of the cohort studies reviewed provided evidence for a dose–response relationship. Overall, as observed with ecological studies, the magnitude of the published risk estimates for kidney cancer was consistently lower than that observed for bladder or urinary organs cancer outcomes.

### Meta-analyses, Model I

**Figure 3:**
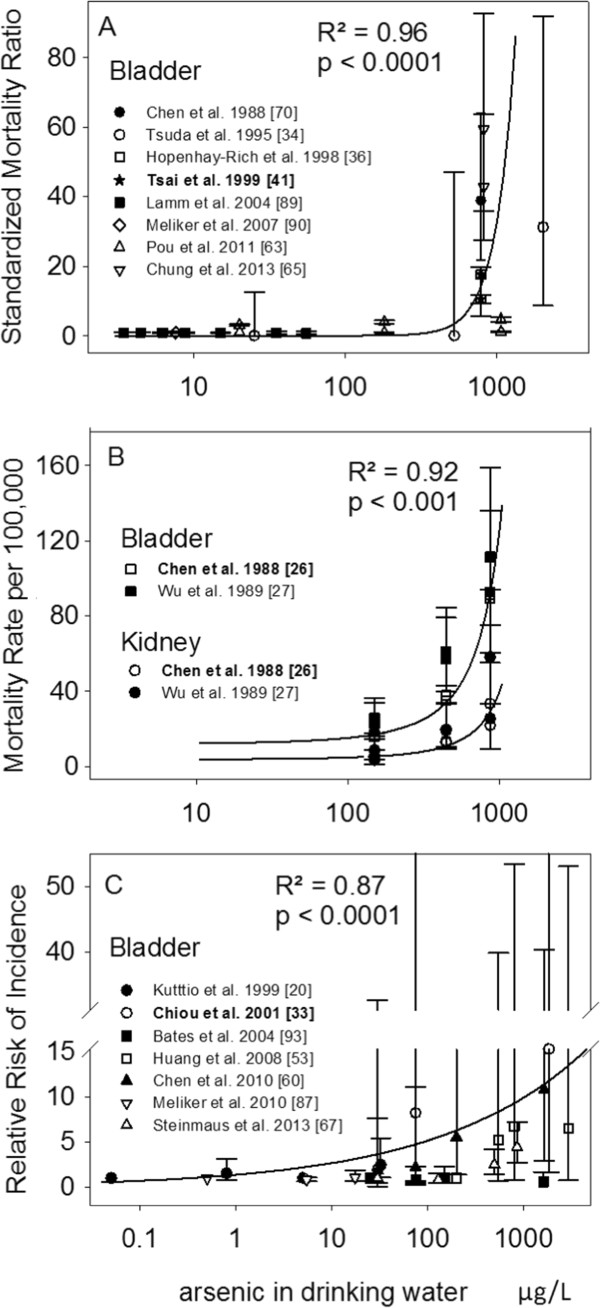
**Published risk estimates for varying levels of arsenic in drinking water in relation to bladder and kidney cancer mortality (A-B) and bladder cancer incidence (C).** Solid lines show the predicted risk from the model fitted values obtained from meta-analyses; referent study for analyses is in bold; R^2^ is the coefficient of determination based upon best fit to distributional assumption. RRs were all adjusted for tobacco smoking. Citation for original publication is in square brackets.

Analyses based on combined epidemiologic data showed an increase in the risk of developing bladder cancer or dying from bladder or kidney cancers with exposure to increasing levels of As in drinking water (Figure 3A-C). Combined bladder cancer SMRs ranged from < 1.0 (As concentration mid-point < 10 μg/L) to 38.8 (As concentration mid-point of 780 μg/L; Figure 3A), showing a significant increase in risk at higher levels of exposure (R^2^ = 0.96, p < 0.0001). Similarly, cancer mortality rates also significantly increased with increased well-water As (Figure 3B; R^2^ = 0.92, p < 0.001). However, the magnitude of the association was three times greater in those dying from bladder cancer relative to those dying from kidney cancer (p < 0.0001). Bladder cancer mortality rates ranged from 15.7 (As mid-point of 150 μg/L) to 91.5 per 100,000 persons (As mid-point of 870 μg/L); kidney cancer mortality rates ranged from 5.4 (As mid-point of 150 μg/L) to 58.0 per 100,000 persons (As mid-point of 870 μg/L). Combined RRs for bladder cancer incidence studies, ranged from 1.0 (As mid-point of 5 μg/L) to 15.3 (As mid-point of 1,845 μg/L) and also indicated a statistically significant increase in risk with increasing well-water As (Figure 3C; R^2^ = 0.87, p < 0.0001). Predicted incidence risk of for bladder cancer increased 2.7 [1.2 – 4.1]; 4.2 [2.1 – 6.3] and; 5.8 [2.9 – 8.7], in those drinking water contaminated with 10 μg/L; 50 μg/L and; 150 μg/L of As, respectively.

### Meta-analyses, Model II

**Figure 4:**
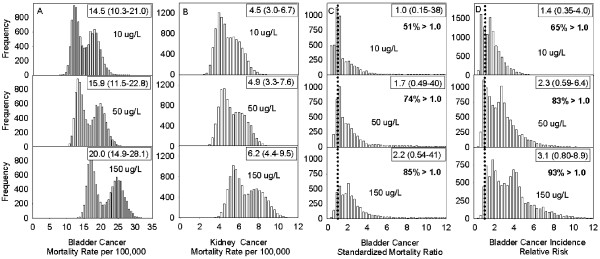
**Distribution of predicted cancer risk estimates (A-B: mortality rates for bladder and kidney cancers; C: standardized mortality ratio for bladder cancer; D: incident relative risk for bladder cancer) at three levels of arsenic concentrations (10, 50 and 150 μg/L) in drinking water.** Distributions were obtained from a bootstrap randomization of the fixed effects arsenic-risk models which were parameterized as a function of logged arsenic and the study from which the data were derived. A total of 10,000 randomizations were used.

The robustness of the effect size at 10, 50 and 150 μg/L of As in drinking water for all three reported outcomes (mortality rates, SMR, RR) was assessed with Model II. The predicted risk derived from the bootstrapped randomizations (Figure 4A-D) confirms the non-linear increase in both bladder and kidney cancer mortality and in bladder cancer incidence with increasing levels of As in drinking water which was observed with Model I. However, the magnitude of the effect size for bladder cancer incidence (Figure 4D) was about 50% lower than those of Model I for exposure to 10, 50 and 150 μg/L of As in drinking water: 1.4, 2.3 and 3.1(Model II) versus 2.7, 4.2 and 5.8 (Model I; Figure 4D). For bladder cancer mortality, the median SMR increased from 1.0 to 1.7 and 2.2 at 10, 50 and 150 μg/L, respectively. For both bladder and kidney cancers, mortality rates at 150 μg/L was about 30% greater than those recorded at 10 μg/L (Figure 4A-C). Although, these effect sizes were not statistically significant, they did follow a dose–response relationship across all outcome measures. In addition, 51% and 65% of the probability density distribution in predicted SMRs and RRs, respectively, fells above 1.0 (no risk) at the lowest exposure benchmark of 10 μg/L, with these proportions increasing to 74% and 83% for SMR and RR at levels of 50 μg/L.

## Discussion

### Summary of findings

This review evaluated 40 studies reporting on the association between As in drinking water and urinary tract cancers. Evidence supporting an increased risk of developing, or dying from, bladder cancer as a result of exposure to As in drinking water was obtained from 28 studies from Taiwan, Chile, Argentina, Japan and Finland. Furthermore, evidence supporting an increased risk of developing, or dying from, kidney cancer due to As in drinking water was obtained from 10 studies from Taiwan and Chile. The risk associated with kidney cancer was consistently of lower magnitude than that reported for bladder cancer outcomes.

Twenty of the 40 studies reviewed were ecological by design, not accounting for potential confounders and with As exposure assigned using well water concentration from geographic or other grouped measurements, which could have resulted in the misclassification of exposure. However, the majority of these studies focused on highly exposed populations where the magnitude of the effects reported was so high that potential confounding or misclassification bias could not fully explain the associations.

Tabulated risk estimates from studies assessing exposure from As in well/tap drinking water, were generally measured within a limited range of As concentrations and varied across, and within regions, even in areas where similar concentrations of As had been measured. Differences in exposure (e.g. As species, timing and duration of exposure) [52] and population characteristics (e.g. genetic variations, lifestyle habits–smoking, diet etc.) have been suggested to contribute to differences in inter-individual susceptibility [52,102,103]. Thus, the methodological limitations of the studies reviewed, including study design, study quality (e.g. level of exposure assessment, lack of adjustment for potential confounders or effect modifiers such as age, sex, cigarette smoking, may have influenced the magnitude of the associations reported. For example, some case–control studies reporting on low exposure levels noted a significant association only among smokers [20,31] and of the cohort studies carried out in Taiwan, those adjusting for such covariates [33,53,60] reported risk estimates three to fourfold lower than ecological studies that did not [24,26].

### Meta-analysis of arsenic in drinking water and the risk of developing bladder or kidney cancers

The analyses of combined risk estimates presented in this review allowed for the examination of the association between cancer outcomes (i.e. mortality and incidence) – independently, and As exposure over a broader and more continuous range of As concentrations. After adjusting for differences in unaccounted bias associated with each study, the results showed that exposure to increasing levels of As in drinking water was significantly associated with an increased risk of bladder and kidney cancer mortality and bladder cancer incidence, regardless of the measure of association employed (i.e. mortality rate, SMR, RR; Model I). Risk estimates obtained from fitted values from Model I showed that people exposed to drinking water contaminated with 10 μg/L of As had more than a twofold increased risk of developing bladder cancer (2.7 [1.2 – 4.1]); those exposed to 50 μg/L and 150 μg/L were expected of have a four- (4.2 [2.1 – 6.3]) and six fold (5.8 [2.9 – 8.7) increase in risk, respectively– relative to the meta-analyses referent group (the general population of Taiwan). Sub-analyses focusing on low-level exposure (≤ 150 μg/L) confirmed the trend, although the effect was slightly reduced at the 150 μg/L exposure level (10 μg/L, RR: 2.8 [1.3 – 4.3]; 50 μg/L, RR: 3.7 [1.7 – 5.7]; 150 μg/L, RR: 4.5 [1.8 – 7.2]). A near six fold increase in bladder cancer risk was also observed by Chen et al. [60] in northeastern Taiwanese residents exposed to levels of As in drinking water ranging between 100–299.9 μg/L (RR: 5.5 [1.4 – 22.0]). However, predicted risks for people exposed to 10 and 50 μg/L were about half of those obtained with Model I but comparable to those of Model II (Figure 4D; see also Chiou et al. [33] for a doubling of risk between 50-100 μg/L). Of note, a recent review reporting on low-level As exposure in drinking water and bladder cancer did not support a significant association [56]. However, their findings were based on a meta-analytical approach that combined incidence and mortality outcomes, and studies using different metrics of exposure (e.g. As in toenails, well water, cumulated etc.), which possibly introduced statistical noise thereby attenuating the summary estimate (risk) towards the null. In this review, risk estimates derived from mortality were smaller than those of incidence data (Figure 4C-D). This possibly reflected patterns of prognosis [104], but perhaps more so, reduced statistical power due to misclassification as eight of the nine studies included in the meta-analyses of SMRs assessed exposure at the group-level, whereas all studies included in the analyses of the incidence data used individual-level measurements or estimations of As in drinking water.

The precise magnitude of excess cancer risk associated with drinking water containing As has been difficult to establish, especially in populations exposed to moderate to low As-levels. A major issue relates to the misclassification of As exposure arising from uncertainties in assessing exposures during the disease-relevant exposure period, which, for As, may extend many decades prior to diagnosis. These uncertainties relate to population mobility, characterization of drinking water sources, assignment of water As concentrations to subjects over time, assessment of fluid intake rates, assessment of dietary As intake, a likely major contributor to exposure in areas of low As-levels [103,105], and difficulties in measuring actual levels of As in drinking water as opposed to relying on estimated levels [56]. Such uncertainties lead to bias which typically results in an underestimation of the true risk— a risk that can be small but still biologically significant.

These uncertainties also act to increase the variability in the distribution of both the measured (e.g. Figure 3) and consequently, the predicted (e.g. Figure 4) risks, weakening the statistical significance of the risk estimate. Studies using biomarkers of exposure offer perhaps a way to reduce such uncertainties that create exposure misclassification. However, rather than limiting the dialogue around As-related health effects to a significance level, perhaps more informative is the high probability that a large proportion of people may be at elevated risk of dying from (Figure 4C, 51% probability) or being diagnosed with bladder cancer (Figure 4D, 65% probability), even at exposure levels as low as 10 μg/L. In this review, we estimate that with exposure to 50 μg/L of As in drinking water there is a 83% probability for an elevated risk of developing bladder cancer and a 74% probability of elevated mortality. (Figures 4C, 4D). Yet, hundreds of millions of people worldwide rely upon drinking water containing As at these concentrations and consider them to be safe [3,69].

### Limitations and strengths

This review has some limitations. First, the search strategy was limited to computerized databases which could preferentially include studies with statistically significant findings [106,107]. While this is a concern, we are confident that publication bias was possibly minimal as a third of the studies included in this review presented non-significant results. Second, the analyses of combined risk estimates were limited to studies providing specific point estimates of As in drinking water, the most common metric of exposure reported. This selection reduced the number of studies eligible for meta-analyses but minimized heterogeneity associated with other exposure metrics such as cumulative As exposure or As concentrations in toenails or urine; two measures linked to population/individual-dependent factors (e.g. years of exposure, cumulated volume of contaminated water ingested, metabolic capacity etc.). Third, analyses were performed independently for studies reporting on different outcomes (i.e. cancer incidence vs. cancer mortality) and different measures of association (i.e. mortality rate, SMR, RR). This stratified approach reduced the statistical power required to analyze the combined data by sex and/or smoking status; the latter being an important effect modifier in the cancer-As relationship. Studies supporting a higher risk among ever smoker are growing in number and so predicted risks presented in this review may be conservative for populations with a high proportion of ever smokers.

Nonetheless, this review has important strengths. First, its broad scope allowed for the inclusion of 30 years of publications and a wide range of exposure from which combined analyses could be performed. Second, the use of a sensitive search strategy ensured a high level of search completeness. Third, while the independent analyses of incidence and mortality outcomes was presented as a limitation in terms of statistical power, it likely minimized possible ascertainment bias and exposure misclassification issues. This is because mortality data are generally less precise than incidence data and the survival rate for bladder cancer is relatively high. In addition, if survival for bladder cancer patients is related to As exposure, then mortality studies could be at greater risk of being confounded compared to incidence studies [104]. Furthermore, exposure in mortality studies is often derived from aggregate data which are more prone to misclassification and bias. Finally, this review updates and complements previously published work, but also provides data which quantifies the risk of developing bladder cancer at varying levels of As exposure, including that observed at lower levels exposure.

## Conclusions

Epidemiological studies provide extensive evidence in support of a causal association between exposure to higher levels of As concentrations in drinking water and the risk of developing or dying from bladder cancer, although the thresholds at which health effects develop remain uncertain at lower levels of As exposure in drinking water. Evidence in support of an increased risk of dying from kidney cancer with exposure to As is also accumulating, but studies reporting on incidence are lacking.

The results of the meta-analysis were consistent with the generally observed findings from the full body of literature reporting on bladder and kidney cancer outcomes and As-exposure. They also confirmed patterns of dose-responses within exposed populations and quantified the evidence for potential health effects at the lower end of the exposure curve where most uncertainties remain. This meta-analysis suggests that populations exposed to 150 μg/L As in drinking water may be increasing their risk of dying from bladder or kidney cancer by 30% relative to those exposed to 10 μg/L. In addition, populations exposed to As concentrations as low as 10 μg/L in drinking water, (which corresponds to the WHO provisional guideline), may be doubling their risk of developing bladder cancer, or at the very least, increase it by about 40% compared to the unexposed populations included in the meta-analyses.

Thus, with the large number of people likely exposed to As in drinking water at the lower range of concentrations throughout the world, we suggest that the public health consequences of As in drinking water may be substantial. And as such, the current advisory limit for concentration of As in drinking water should be reviewed as well as policies on the promotion and support of household water arsenic remediation activities. Further studies focusing on populations exposed to low As concentrations with exposure measured at the individual level (e.g. biomarker studies), are required to confirm the observed health effect suggested in this review.

## Abbreviations

WHO: World Health Organization; As: Arsenic; PubMed: Public/Publisher MEDLINE; BMI: Body mass index.

## Competing interests

The authors declare that they have no competing interests.

## Authors’ contributions

NSJ conducted the literature search for this review, specified the inclusion and exclusion criteria, abstracted published data, modeled combined risk estimates, constructed tables and figures, drafted and revised the manuscript; LP and TD supervised the review, reviewed the article critically for important intellectual content and provided important assistance in the interpretation. PB provided intellectual content and statistical advice to carry the meta-analyses. All of the authors gave final approval.
